# Medical students’ perspectives on earning opportunities of self-employed physicians — realistic and relevant for the process of career choice?

**DOI:** 10.1186/s12909-020-1950-y

**Published:** 2020-02-10

**Authors:** Tobias Deutsch, Alexander Heine, Stefan Lippmann, Anne-Kathrin Geier, Alexander Bauer, Thomas Frese

**Affiliations:** 1 Department of General Practice, Faculty of Medicine, University of Leipzig, Leipzig, Germany; 2 Institute of General Practice and Family Medicine, Martin-Luther-University Halle-Wittenberg, Halle/Saale, Germany

**Keywords:** Career choice, Medical students, Primary health care, General practice, Germany, Income, Earning opportunities

## Abstract

**Background:**

Several studies report a substantial impact of financial considerations on the process of specialty choice and the willingness to establish one’s own practice. In Germany, reliable information on self-employed physicians’ earning opportunities is basically available, but not easily accessible and understandable for medical students. Misperceptions might contribute to recruitment problems in some fields, particularly in general practice. In order to identify a possible need for action, we investigated current German medical students’ level of information regarding future earnings, and whether net earnings of general practitioners and other physicians working self-employed are estimated realistically. Additionally, we explored students’ self-assessments regarding the extent of the impact of expected earnings on their personal career choice process.

**Methods:**

We conducted a cross-sectional questionnaire survey among fourth year (of six) medical students at one medical school (Leipzig). The participants estimated the net earnings of different physicians working self-employed. These estimations were compared with actual earnings data derived from a large German practice panel.

**Results:**

Response rate was 73.6% (231/314). The participants’ mean age was 24.9 years and 59.1% were women. On a 10-point scale ranging from 1 = ‘no influence’ to 10=‘very big influence’, 92.6% of the participants described at least some (≥2) influence of earning expectations on their career choice process, and 66.2% stated this influence to be 5 or higher. Every fourth student (26.4%) would rather or definitely reject a certain specialty because of expected low earning opportunities. While 60.4% had already thought about future earnings, only 26.8% had obtained concrete information. Compared with the data derived from the practice panel, the participants substantially underestimated the earning opportunities in self-employed settings, including general practice (median: 4500 vs. 6417€). However, depending on the single estimations, between 87.7 and 95.6% of the students stated they were ‘rather uncertain’ or ‘very uncertain’ regarding their estimations.

**Conclusions:**

Despite confirming a relevant impact of financial considerations on career choice, German fourth year medical students are not well informed about earning opportunities in self-employed settings. Providing easily understandable information could enhance transparency and might help students to consider financial issues of career choice on a realistic basis.

## Background

To find responses to recruitment problems in primary care and particularly in general practice, many studies worldwide have tried to elucidate the complex process of career decision making in medicine [1]. A wide range of factors influencing specialty choice [2–5], as well as the decision to establish one’s own practice [6], have been identified. These factors include for example socio-demographic characteristics, personal and professional interests and preferences, career considerations at matriculation, experiences during medical school, perceived specialty characteristics, as well as family and lifestyle considerations [2–6]. One factor that has been reported with high consistency is the relevant impact of financial considerations [2–4, 6–8]. A recent study from Israel particularly emphasized the influence of a ‘reasonable income to lifestyle ratio’ on young physicians’ career choice [9]. These findings are in line with results of a former study of our own research group, showing that the expectation of inadequate earning opportunities, in general or in relation to workload, was among the most frequently mentioned motives of German graduates to reject a career as a general practitioner (GP) [10]. However, according to data from a large German practice panel annually analysing the economic situation of physicians working self-employed in their own practices [11], the perception of a general and severe financial disadvantage for GPs compared to other specialists working self-employed or in hospital settings appears to be rather unfounded.

In Germany, undergraduate medical education takes 6 years and is followed by a residency of 4 to 6 years (depending on the speciality) to qualify as a clinical specialist (German “Facharzt”). To become a general practitioner 5 years of residency have to be completed including rotations in the hospital as well as in outpatient care. Generally, the German health care system is divided into an inpatient and an outpatient sector. While inpatient care is provided by physicians employed by hospitals, outpatient care is mainly provided by self-employed physicians in their own practices. While physicians working in hospitals receive a salary, self-employed physicians in their own practices are remunerated mainly based on a fee-for-service system financed by patients’ statutory or private health insurances. The vast majority of the patients (> 90%) are insured via the (mandatory) statutory insurance plan [12].

It has been recently stated by Merk & Merk, both experts in valuation of enterprises in German healthcare, that young physicians might assess the attainable earnings in self-employed settings frequently based on faulty information and misperceptions [13]. Considering the above-mentioned evidence underlining the relevant impact of financial considerations on the career choice process, it seems to be likely that misconceptions regarding earning opportunities in self-employed settings and particularly in general practice would have the potential to contribute to a certain extent to recruitment problems in the field. However, respective misperceptions might be overcome by providing sound information. In order to identify a possible need to provide such information already in the course of undergraduate medical education, this study aimed to explore medical students’ level of information regarding future earnings and particularly regarding the earnings of physicians working self-employed in their own practice. We wanted to investigate if students have obtained concrete information on future earnings, and which sources of information they have used. As a central outcome it was of interest whether medical students have realistic conceptions of the monthly net earnings of physicians working self-employed and to what extent students’ estimations possibly differ from reality. Considering the pressing recruitment problems in general practice threatening the nationwide availability of nearby outpatient care, we were particularly interested in the students’ estimations of the earnings of GPs working self-employed. Furthermore, to be able to assess the direct importance of our results within our sample, we additionally wanted to explore current German medical students’ detailed self-assessment of the impact of expected earning opportunities on their personal career choice process, although the impact in general is known from literature.

## Methods

### Sampling and design

The present data are based on a cross-sectional questionnaire survey which was conducted at the University of Leipzig, Faculty of Medicine, in May 2017. The anonymous questionnaire was provided to fourth year medical students immediately prior to a mandatory written test following the general practice lecture series. Participation was on a voluntary basis after being comprehensively informed about the study and its purpose.

### Questionnaire

We used a self-developed questionnaire created by a multidisciplinary team consisting of a general practitioner, a general practice resident, a psychologist, and an economist. It contained items addressing socio-demographics, career preferences, previous search for information on future earnings, the estimated influence of earning expectations on the own career choice process, estimations of the net earnings of physicians working in different self-employed settings, and the students’ self-rated confidence when estimating the respective earnings. Net earnings were introduced as the personally available amount of money at the end of a month after all statutory deductions (for a fulltime work scenario). Prior to the survey, the questionnaire was pre-tested with two medical students in advanced study years (representing the target group) to ensure comprehensibility, usability, and face validity. The pre-testing procedure was inspired by the method of concurrent think aloud (CTA) and led to minor adjustments regarding content and form in the final version. An English translation of the questionnaire items is given in Additional file 1.

### Calculation of the comparable figures regarding self-employed physicians’ earnings

The comparable figures regarding the earnings of physicians working self-employed in their own practice were derived from data provided by the ZI (Zentralinstitut für die kassenärztliche Versorgung = Central Research Institute of Ambulatory Health Care in Germany) [11]. The ZI is the official research institute of the German Federal Association of Statutory Health Insurance Physicians. The “ZI practice panel” [11] is a representative panel of 5006 practices of different specialities, including 749 GPs. This panel annually analyses the economic situation of self-employed physicians for official purposes. In 2015, the financial analysis was based on 4173 practices including 659 GPs. As a central outcome, the panel discloses the annual net profit per practice owner before income tax and mandatory health and pension insurance costs.

For a better comparability of these profits to the net earnings of employed physicians (e.g. in hospitals) the ZI “annual net profit” needed to be modified. In accordance with recommendations of the ZI [11], we deducted the income tax, and official mandatory health and pension insurance costs. The assumptions for our calculation were strictly conservative (unmarried taxpayer, no children, maximum rates in health and pension insurance). The resulting “net earnings” can be understood as the money available for private purposes.

### Statistics

Data was analyzed using IBM SPSS Statistics 24 for Windows. Considering missing values for single items frequencies were presented as %_valid_ (n_absolute_/n_valid_). Continuous variables were presented as mean ± standard deviation (SD) complemented by median and quartiles, if appropriate. In addition to descriptive statistics, Wilcoxon signed-rank test (related measures) as well as Mann-Whitney U-Test (independent group comparisons) were used to analyze differences in central tendency. Statistical significance was assumed for *p* < 0.05. If appropriate, mean differences including 95% confidence intervals (CI) were reported additionally.

## Results

Out of 314 students taking part in the general practice test, 231 completed the questionnaire resulting in a response rate of 73.6%. The socio-demographic characteristics of the sample are presented in Table 1. General practice was the currently favored career option for 8.7% (20/231) of the participants and 33.8% (78/231) at least considered it as an option.

**Table 1:** Sample characteristics

Variable	valid (*N*)*	*N* (%)**
Age [mean ± SD]	231	24.9 ± 3.4
Female	230	136 (59.1)
In a relationship	217	116 (53.5)
Has children	225	18 (8.0)
At least one parent with higher education degree	228	172 (75.4)
Being a physician’s child	231	49 (21.2)
Family or friends working in general practice	231	77 (33.3)
Mainly grown up in …	229	
big city		69 (30.1)
small town		88 (38.4)
rural area		72 (31.4)
Pre-existing concluded education in a medical vocational education	230	64 (27.8)

* N’s vary due to missing values

** Unless otherwise indicated

**Fig. 1:**
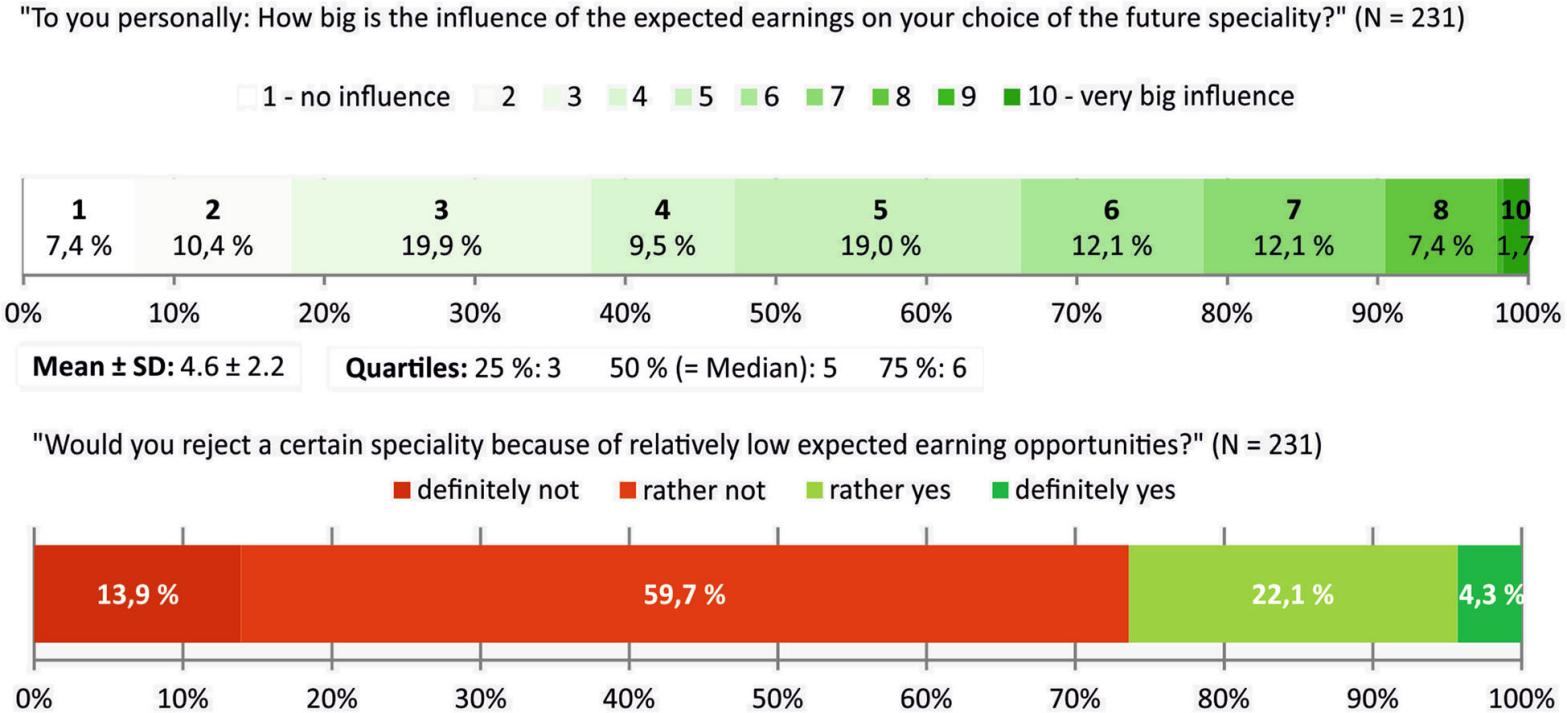
Influence of expected earning opportunities on career choice as perceived by German medical students

On a 10-point Likert item ranging from 1 = ‘no influence’ to 10 = ‘very big influence’ 92.6% of the participants stated that there is at least some (≥2) influence of earning expectations on the personal choice of the future specialization. For 52.8% this influence was 5 or higher. The median response was 5 and the mean response 4.6 ± 2.2. One quarter of students would rather or definitely reject a certain specialty because of relatively low expected earning opportunities (Fig. 1).

**Fig. 2:**
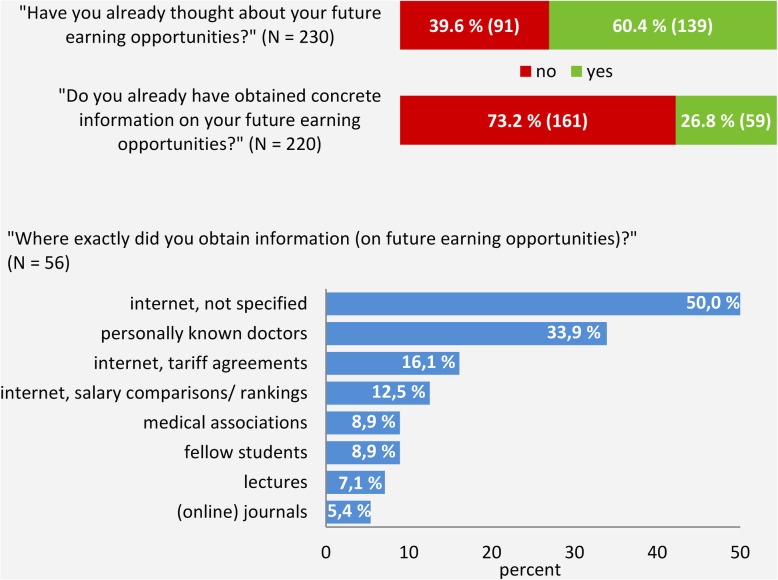
Students’ interest in future earning opportunities and information obtained prior to the study

While three out of five students had already thought about future earnings, only a quarter had obtained concrete information prior to the survey. Among those who had obtained such information the most common sources of information were “internet” (not further specified) as well as personally known doctors (Fig. 2).

**Fig. 3:**
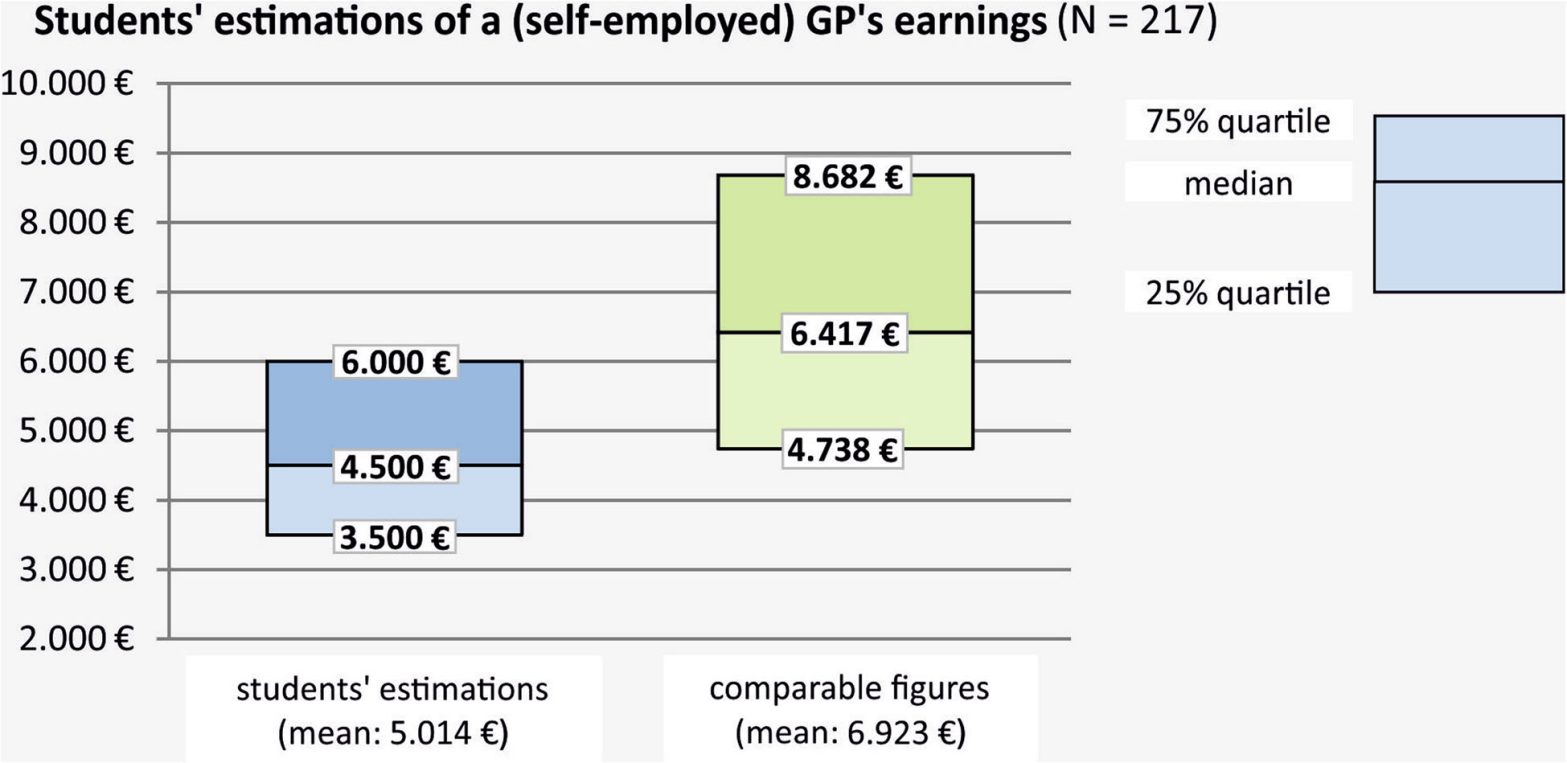
German medical students’ estimations of a GP’s net earnings vs. comparable Figs. (ZI panel)

Approximately nine out of 10 participants stated they were ‘rather uncertain’ or ‘very uncertain’ (compared to ‘rather certain’ or ‘very certain’) to estimate concrete net earnings, ranging between 87.7 and 95.6% for the single estimations. The comparison of students’ estimations of a GP’s net earnings with the data derived from the ZI practice panel revealed that the participating students substantially underestimated GPs’ earnings (Fig. 3). On average, students estimated the net earnings of GPs working in urban areas moderately but significantly higher than the earnings of GPs working in non-urban areas (median: 4700 vs. 4500 €; mean ± SD: 5155 ± 2275 € vs. 4872 ± 2085 €; mean difference MD (95% CI): 283 (146–421); Wilcoxon signed-rank test: *p* < 0.001). We found no significant differences in the earning estimations for GPs working self-employed depending on student characteristics and preferences like gender, the parents’ education degree, a pre-existing concluded education in a medical occupation, general practice as the currently favored career, general practice as a considered career, and the fact that someone had already obtained concrete information on future earning opportunities. However, we found significantly higher estimations for students whose parents are physicians and who have family or friends working in general practice (Table 2).

**Table 2:** Differences in students’ estimations of self-employed GPs’ earnings depending on personal characteristics and preferences

Variable		valid (*N*)*	Estimated GP net earnings in €			p**
			Median	Mean ± SD	Mean difference MD (95% CI)	
Gender	men	88	4500	5137 ± 2150	191 (− 385–767)	0.455
	women	128	4500	4946 ± 2103		
At least one parent has a higher education degree	yes	164	4500	5030 ± 2217	65 (− 606–736)	0.775
	no	50	4500	4965 ± 1753		
At least one parent is a physician	yes	47	5000	5821 ± 2793	1030 (358–1702)	**0.021**
	no	170	4500	4791 ± 1841		
Has family or friends working in general practice	yes	73	4750	5459 ± 2325	671 (79–1263)	**0.039**
	no	144	4500	4788 ± 1979		
Pre-existing concluded education in a medical occupation	yes	61	4500	5138 ± 2086	173 (− 456–802)	0.506
	no	156	4500	4965 ± 2138		
General practice is the currently favored career	yes	19	4500	5566 ± 2239	602 (− 402–1606)	0.254
	no	187	4500	4964 ± 2116		
General practice is a considered career option	yes	74	4500	5032 ± 2105	28 (− 568–624)	0.962
	no	143	4500	5004 ± 2135		
Had already obtained concrete information on future earning opportunities	yes	56	5000	5330 ± 2012	456 (− 185–1702)	0.064
	no	151	4500	4874 ± 2117		

* N’s vary due to missing values

** Mann-Whitney U-Test; *p*-values ≤0.05 are printed in bold

Students who currently favored a career in other specialities than general practice were additionally asked to estimate the earnings of physicians working self-employed in their favored field. These earnings were underestimated as well (mean_students’ estimations_ vs. mean_comparable figures_): internal medicine (*N* = 50; 5188 vs. 10,246 €), paediatrics (*N* = 22; 5180 vs. 6914 €), gynaecology (*N* = 16; 4313 vs. 7374 €), surgery (*N* = 26; 4854 vs. 7304 €), anaesthesiology (*N* = 13; 5531 vs. 7155 €).

## Discussion

### Summary of the main findings

The students in our sample confirmed a relevant impact of earning expectations on their career choice process. While in the fourth study year a majority of the students had already thought about future earning opportunities, only a minority had obtained concrete information. Coincidently, the net earnings of physicians working self-employed, including those of GPs, were significantly underestimated.

### Literature comparison

Our findings regarding the relevance of financial considerations are in line with international study results which reported an influence of income expectations on career decision making in medicine with a high consistency [2–4, 14, 15]. Also, for the German context it has been shown that the perceived financial conditions seem to play a crucial role for specialty choice and the decision of whether to establish one’s own practice or not [6, 7, 10]. The participants in our study used all steps of our 10-point scale to quantify the influence of expected earnings on their personal career choice process, indicating fine inter-individual differences. Our results show that although many medical students in the fourth study year are already dealing with the topic of future earnings, they are not well informed and have no realistic conceptions about earning opportunities in self-employed settings, including differences between specialties and settings like urban and non-urban practice environment. Indications for an underestimation of non-urban GPs’ net earnings were also found among German general practice residents [16]. This is in line with the perception of experts in healthcare entrepreneurship, who stated that even physicians after graduation and young specialists tend to have limited knowledge about attainable earnings in self-employed settings [13].

One factor contributing to this information gap may be a lack of easily accessible, reliable, easily understandable, and comparable statistics regarding the attainable earnings of physicians working in self-employed settings in Germany [17]. The reliability of information from many online career portals can be questioned because of frequently lacking information on the age, origin, and nature of the data. Official statistics are available from the National Association of Statutory Health Insurance Physicians (Kassenärztliche Bundesvereinigung, KBV) [18] and the Federal Statistical Office (Statistisches Bundesamt) [19]. However, these statistics are partially incomplete regarding the sum of all gains and costs [20] and without basic economical knowledge it is hardly possible to get an idea of single physicians’ monthly net earnings [13, 17]. But this in particular would be helpful for a sound comparison with the income of employed physicians, which is easily accessible via official websites containing tariff tables and respective calculators. Furthermore, the frequently communicated means do not adequately reflect the wide range of earnings between different practices within the same specialty, which would be necessary to assess earning opportunities in a sense of ‘attainable earnings’ in a field. The ZI practice panel [11], which was used for the calculation of comparable figures in this study (see methods section), can be seen as the best available database for Germany. The data are based on the annual net profits provided by the physicians’ tax consultants, considering all gains and costs. Furthermore, the ZI provides quartiles regarding the physicians’ earnings in addition to means to reflect the wide range of attainable earnings in a field. Unfortunately, also for these data a simple and comparable preparation of the relevant information, easily understandable for medical students is missing.

In our study, students whose parents are physicians and who have family or friends working in general practice estimated the earning opportunities in a GP practice higher than their counterparts, certainly due to personal insights and impressions. As these insights are reserved to a minority and even these students still underestimated the earnings, measures should be taken to provide realistic and understandable information to all medical students.

There is broad evidence that for a majority of the students career choice is a long process accompanying undergraduate education and residency, and of course, influence factors and considerations made by the students are manifold (see introduction section) [2, 3, 5, 21]. However, it has been shown that money is one important issue. Our results support the hypothesis that in Germany many students and young physicians have misperceptions concerning the earning opportunities in self-employed settings. To increase their interest in primary care careers and the establishment of their own practices it seems advisable to correct such inaccurate representations [22]. This should be done early enough during medical school to avoid deselection of careers based on misperceptions. Better information for medical students about finances in self-employed settings has been already suggested [6]. Previous studies imply that medical students are open-minded regarding entrepreneurial aspects and business management and they experience respective content as meaningful for their education [23]. 

### Strengths and limitations

This investigation discloses practical implications to positively influence a process of informed career choice among medical students and young physicians. The sample size and the acceptable response rate support the informative value of the data. As a first limitation it can be critically discussed that we have asked medical students in the fourth study year about a topic that might become more relevant shortly before graduating (in Germany after 6 years of studies). With the chosen cross-sectional design, we were not able to evaluate whether the revealed misconceptions persist in graduates. However, as medical career choice must be understood as a continuous process during the whole time span of medical studies and residency [2, 3, 21] it could be helpful to fight misperceptions regarding relevant influence factors as early as possible. Furthermore, it has been shown by other studies that even residents still underestimate self-employed physicians’ earning opportunities [16]. A second limitation might be that we have investigated medical students from only one faculty of medicine, which must be considered when generalizing the findings. Also, the fact that some results are relatively specific for the German context, at least regarding absolute numbers (earnings), limits the generalizability. As a further limitation it could be discussed that we didn’t carry out an initial pilot study, which might have led to further modifications of our questionnaire. However, we pre-tested the questionnaire with two students representing the target group to ensure comprehensibility, usability, and face validity (see methods section). Finally, it should be noted that the calculation of our comparable figures regarding self-employed physicians’ net earnings based on data of the ZI practice panel was following rigid presuppositions. Consequently, the comparable figures do not consider variations associated with individual circumstances. However, as our presuppositions have been quite conservative, it can be assumed that the resulting net earnings are rather under- than overestimated.

## Conclusions

This study contributes to research on medical students’ career choice by disclosing a new aspect: While it was known that financial considerations have a relevant impact on the process of career choice, our results show that these considerations may be strongly biased by misperceptions and a lack of information. Although a majority of the students in our sample confirmed a considerable influence of earning expectations on their career considerations, they were not well informed and underestimated earning opportunities in self-employed settings, including general practice. This might negatively affect the attractiveness of a respective career as well as the consideration of establishing one’s own practice in the future. Providing easily accessible, understandable, and comparable information on earnings of self-employed physicians, considering working conditions in different settings, could enhance transparency and might help students to consider financial issues of career choice on a realistic basis. We have described that for the German context realistic information on earning opportunities in self-employed settings is basically available but should be restructured and spread more widely to adequately inform medical students. One approach may be to integrate the topic into the undergraduate curriculum at an appropriate time, e.g. in the form of a short workshop dealing with the income structure, workload and job satisfaction of physicians working in self-employed settings. Further studies could examine students’ interest in respective learning content and its effect on career considerations. More generally, our results are of interest for persons who provide career guidance or mentoring to medical students, medical graduates, and residents, as well as for institutions trying to convince young physicians to establish their own practices (e.g. to secure medical care in underserved areas). For countries where no realistic information on earnings in self-employed settings is available, efforts could be made to elucidate this ‘black box’ and to monitor reliable data to allow medical students and young physicians informed decisions.

## Supplementary information

**Additional file 1.** English translation of the questionnaire items analysed in this study.

## Data Availability

The datasets used for the current study are available from the corresponding author on reasonable request.

## Abbreviations

CTA: Concurrent Think Aloud
GP: General Practitioner
KBV: Kassenärztliche Bundesvereinigung (National Association of Statutory Health Insurance Physicians)
SD: Standard Deviation
ZI: Zentralinstitut für die kassenärztliche Versorgung (Central Research Institute of Ambulatory Health Care in Germany)
