# Supplementary material for: Medical students’ perspectives on earning opportunities of self-employed physicians — realistic and relevant for the process of career choice?
Source: BMC Med Educ. 2020 Feb 10;20:42. doi: 10.1186/s12909-020-1950-y (PMC7011452; doi:10.1186/s12909-020-1950-y)
Supplement: Supplementary file 1 — Additional file 1. English translation of the questionnaire items analysed in this study. [file 12909_2020_1950_MOESM1_ESM.docx]

**Additional File 1: English translation of the questionnaire items analysed in this study**

| **Personal information** | | | | | | | | | | | | | | | | | | | | | | | | | |
| --- | --- | --- | --- | --- | --- | --- | --- | --- | --- | --- | --- | --- | --- | --- | --- | --- | --- | --- | --- | --- | --- | --- | --- | --- | --- |
| Age: |  | \|__\|__\| years | | | | |  | Sex: | | | | ○ | | male | | | | ○ | female | | | | | | |
| Are you in a relationship? | | | ○ | yes | ○ | no |  | Do you have children? | | | | ○ | | yes | | | | ○ | no | | | | | | |
| Do you have at least one parent with a higher education degree? | | | | | | | | | | | | | | | | ○ | yes, one or both | | | | | ○ | no, none | | |
| Is one of your parents a physician? | | | | | | | | | | | | | | | | ○ | yes | | | | | ○ | no | | |
| Do you have family or friends working in general practice? | | | | | | | | | | | | | | | | ○ | yes | | | | | ○ | no | | |
| Where did you mainly grow up? | | | | | | | | | ○ | big city | | | | | ○ | small town | | | | ○ | rural area | | | | |
| I have a qualification in a medical vocational education. | | | | | | | | | | | ○ | | yes, ___________________________ | | | | | | | | | | | ○ | no |

| **Career preferences** | | | | | | | |
| --- | --- | --- | --- | --- | --- | --- | --- |
| My currently favoured career (specialty) is: | | ○ | general practitioner | ○ | other specialist:  ________________________ | ○ | don't know yet |
| Many medical students have not definitely chosen their future specialty, but consider a number of options. Please sort the specialties you are currently considering as possible career options in a descending order (max. 4 specialties): | | | | | | | |
|  | First choice: | ______________________________________________________________ | | | | | |
|  | Second choice: | ______________________________________________________________ | | | | | |
|  | Third choice: | ______________________________________________________________ | | | | | |
|  | Fourth choice: | ______________________________________________________________ | | | | | |

| **Influence of financial considerations on specialty choice** |
| --- |

| Have you already thought about your future earning opportunities? | | | ○ | yes | ○ | no |
| --- | --- | --- | --- | --- | --- | --- |
| "Do you already have obtained concrete information on your future earning opportunities? | | | ○ | yes | ○ | no |
|  | *Where exactly did you obtain information (on future earning opportunities)?* | | | | |  |
|  |  |  | | | |  |
|  |  |  | | | |  |
|  |  |  | | | |  |
|  |  |  | | | |  |

| To you personally: How big is the influence of the expected earnings on your choice of the future speciality? | | | | | | | | | | | |
| --- | --- | --- | --- | --- | --- | --- | --- | --- | --- | --- | --- |
| *no influence* | ➀ | ➁ | ➂ | ➃ | ➄ | ➅ | ➆ | ➇ | ➈ | ➉ | *very big influence* |
| Would you **reject** a certain speciality because of relatively low expected earning opportunities? | | | | | | | | | | | |
| ○ definitely not | | | ○ rather not | | | ○ rather yes | | | ○ definitely yes | | |

| **Earning estimations** |
| --- |
| **Please estimate the monthly net earnings (for private use after all statutory deductions) of the following physicians (full-time job). Please indicate in each case how confident you are regarding your estimation.** |

| **Estimation of the monthly net earnings** | | | **How confident are you regarding your estimation?** | | | |
| --- | --- | --- | --- | --- | --- | --- |
|  | | | very uncertain | rather uncertain | rather certain | very certain |
| **General Practitioner** | | | | | | |
| … | working self-employed (own practice), small-town/rural area | ___________ € net/ month | ○ | ○ | ○ | ○ |
| … | working self-employed (own practice), big city | ___________ € net/ month | ○ | ○ | ○ | ○ |
| **Specialist in the currently favoured specialty (first choice, as stated above!)**  *(If general practice is the favoured specialty, you do not have to complete this a second time.)* | | | | | | |
| … | working self-employed (own practice), small-town/rural area | ___________ € net/ month | ○ | ○ | ○ | ○ |
| … | working self-employed (own practice), big city | ___________ € net/ month | ○ | ○ | ○ | ○ |
